# Endocannabinoids mediate spike-timing dependent potentiation and depression: a model-based experimental approach

**DOI:** 10.1186/1471-2202-14-S1-O1

**Published:** 2013-07-08

**Authors:** Yihui Cui, Vincent Paille, Bruno Delord, Stéphane Genet, Elodie Fino, Laurent Venance, Hugues Berry

**Affiliations:** 1 Team Dynamic and Pathophysiology of Neuronal Networks, Center for Interdisciplinary Research in Biology (CIRB), CNRS UMR7241/INSERM U1050, College de France, 75005 Paris, France; 2 Memolife Laboratory of Excellence and Paris Science Lettres Research University, Paris, France; 3 University Pierre et Marie Curie, ED 158, 75005 Paris, France; 4 Institute of Intelligent Systems and Robotics (ISIR), 75005 Paris, France; 5 Team Beagle, INRIA Rhone-Alpes, 69603 Villeurbanne, France; 6 University of Lyon, LIRIS UMR5205, 69621 Villeurbanne, France

## 

Activity-dependent long-term potentiation (LTP) and depression (LTD) of synaptic strength underlie multiple forms of learning and memory. Endocannabinoids (eCBs) have consistently been described as mediators of short- or long-term synaptic depression through the activation of the endocannabinoid-type-1 receptor (CB1R) or the transient receptor potential vanilloid-type-1 (TRPV1). Here we investigated whether eCBs could also promote long-term potentiation, an essential requirement for eCBs to be a genuine bidirectional system and to fully encode for learning and memory.

To this aim, we combined *in vitro *spike timing-dependent plasticity (STDP) protocols in rodents and a biophysical model of the signaling pathways likely to be involved. The model describes the temporal dynamics of three main signaling systems: the postsynaptic NMDAR-CaMKII pathway (adapted from [1]), the postsynaptic mGluR-PLCβ system (adapted from [2]) as well as postsynaptic eCB synthesis and subsequent activation of postsynaptic TRPV1 and presynaptic CB1R in a retrograde fashion. Using the model to drive the experiments, we uncovered the existence of an eCB-mediated spike-timing dependent potentiation (eCB-LTP). This eCB-LTP is homosynaptic, astrocyte-independent and expressed in young and adult animals and across various brain regions (cortex and striatum), supporting its role as a widespread signaling system for spike-based plasticity. We deciphered the signaling pathways (pre- and postsynaptic receptors and enzymes) involved in this new form of plasticity and demonstrated that eCB plasticity has a postynaptic induction and a presynaptic maintenance. On the postsynaptic side, our results show that the dynamics of free cytosolic calcium is a key element for eCB-LTP induction. eCB-LTP is triggered when eCB transients reach sufficiently high levels. Since the enzymes that synthetize eCBs are calcium-activated, eCB-LTP induction requires large levels of cytosolic calcium. On the presynaptic side, eCBs encode for bidirectional plasticity via a triad composed of eCB levels, presynaptic PKA and presynaptic CaN: intermediate eCB levels promote presynaptic CaN activity, that yields eCB-LTD, whereas large eCB amplitudes favor presynaptic PKA activity, which leads to eCB-LTP. Both effects are predicted to rely on the inhibition exerted by activated CB1R on presynaptic adenylate cyclase and P/Q-type voltage-gated calcium channels. Moreover, we show that eCB-LTP and eCB-LTD can be induced sequentially in the same neuron, depending on the cellular conditioning paradigm. Therefore, our results demonstrate that eCBs, just like glutamatergic or GABAergic signaling, form a generic system able to encode for bidirectional plasticity and capable of genuine homeostasis.

Lastly, we found that eCB-LTP is triggered by very few paired spikes (5 to 10 post-pre spikes at 1 Hz are enough). Thus, eCB-LTP provides synapses with a mechanism able to react to the very first occurrences of incoming activity. This ability strongly contrasts with NMDAR-dependent LTP which, in a classical (1 Hz) STDP context, requires the iteration of at least 75-100 paired stimulations to be expressed, at odds with the observations that new associative memories and behavioral rules can be learned within few or even a single trials in mammals (e.g. [3]). Our results suggest that eCB-LTP may represent a neuronal substrate for such rapid learning abilities.
